# Placental Dysfunction and Congenital Heart Disease: Investigating the Placenta‐Heart Axis

**DOI:** 10.1002/pd.70194

**Published:** 2026-06-10

**Authors:** Natalie Lanners, Sarah U. Morton, Katherine Le, Steffy Blanco, Sara Mansoorshahi, Chrystalle Katte Carreon, Christina Ronai, Louise Wilkins‐Haug, Vidhya Annavajjhala, Jacqueline G. Parchem, Tina O. Findley

**Affiliations:** ^1^ McGovern Medical School The University of Texas Health Science Center at Houston Houston Texas USA; ^2^ Department of Pediatrics Harvard Medical School Boston Massachusetts USA; ^3^ Department of Pediatrics Division of Newborn Medicine Boston Children's Hospital Boston Massachusetts USA; ^4^ Department of Pediatrics Division of Neonatal‐Perinatal Medicine McGovern Medical School The University of Texas Health Science Center at Houston Houston Texas USA; ^5^ Department of Pathology Boston Children's Hospital Boston Massachusetts USA; ^6^ Department of Pathology Harvard Medical School Boston Massachusetts USA; ^7^ Department of Cardiology Boston Children's Hospital Boston Massachusetts USA; ^8^ Division of Maternal‐Fetal Medicine and Reproductive Genetics Brigham and Women's Hospital Boston Massachusetts USA; ^9^ Department of Obstetrics Gynecology and Reproductive Biology Harvard Medical School Boston Massachusetts USA; ^10^ Department of Pediatrics Division of Pediatric Cardiology McGovern Medical School The University of Texas Health Science Center at Houston Houston Texas USA; ^11^ Department of Obstetrics Gynecology and Reproductive Sciences McGovern Medical School The University of Texas Health Science Center at Houston Houston Texas USA

## Abstract

**Objective:**

Concurrent development of the placenta and heart during early gestation suggests a shared biological basis for the co‐occurrence of abnormal placentation and congenital heart disease (CHD). This study investigated the association between placental vascular pathology and CHD type.

**Methods:**

A retrospective study at two institutions included CHD (*n* = 521) and unaffected (*n* = 122) infants. CHDs were categorized into three groups (left and right ventricular outflow tract obstruction and mixed lesions) based on the anticipated effect of placental venous return streaming toward the fetal brain. Placental pathological findings were categorized by the Amsterdam criteria. The rate of placental pathology was compared between cases and controls and across the three CHD groups.

**Results:**

CHD had higher rates of maternal vascular malperfusion (MVM) (0.31 vs. 0.05, *p* < 0.001). Comparison within CHD groups demonstrated similar rates of MVM, while fetal vascular malperfusion (FVM) was significantly higher in groups with reduced fetal cerebral oxygenation (*p* = 0.037). MVM was associated with low birth weight (OR = 0.28, *p* < 0.001), and FVM was associated with increased maternal age (OR = 1.07, *p* = 0.037). No significant associations were identified in other placental pathologies.

**Conclusion:**

This study offers valuable insights into the connection between placental dysfunction and CHD, identifying that MVM is significantly associated with CHD development.

## Summary

- What's already known about this topic?
  ◦ Congenital heart disease (CHD) is the most common birth defect worldwide, yet its causes remain poorly understood. Increasing evidence suggests a developmental link between the placenta and fetal heart, as both form concurrently during early gestation. Prior studies have observed placental abnormalities in CHD‐affected pregnancies, but the relationship between specific placental vascular pathologies and CHD phenotypes remains unclear.
- What does this study add?
  ◦ This study's findings strengthen evidence for the placenta–heart developmental axis and provides novel insights into how distinct placental abnormalities contribute to certain CHD phenotypes. Specifically, maternal vascular malperfusion (MVM) is significantly more common in CHD cases compared with unaffected controls, independent of CHD subtype. Fetal vascular malperfusion (FVM) was also found to be increased in CHD subtypes associated with reduced fetal cerebral oxygenation (e.g., left ventricular outflow tract obstruction, transposition of the great arteries).

## 1 Introduction

Congenital heart disease (CHD) is the most common birth defect across the globe, affecting approximately 1% of live births [1]. Genetic risk contributes to approximately 50% of CHD [2, 3]. Teratogens and environmental factors are other significant influences, but the cause of most CHDs remains undetermined [5]. There is growing interest in the role of the placenta in fetal heart development because of a common developmental and physiological link. The placenta and the fetal heart develop simultaneously during gestation, and the embryonic vasculature also creates a direct connection between these two organs [6]. This developmental relationship forms the basis for what has been termed the “placenta‐heart axis” [7]. The placenta performs many critical functions necessary for maternal and fetal health. It provides nutrients, removes waste, produces essential hormones, and provides immune protection. Given its vital role, it is biologically plausible that disruptions in placental function increase the risk of fetal complications. The relationship between certain placental disorders, namely pre‐eclampsia and CHD, is now well‐established. Preeclampsia, a hypertensive disorder of pregnancy, is a leading cause of adverse maternal and neonatal outcomes, including maternal end‐organ damage and fetal growth restriction caused by placental insufficiency compromising blood supply to the fetus [8, 9]. Prior studies have shown that early‐onset and severe pre‐eclampsia is associated with an increased risk of CHD in offspring [10, 11]. Conversely, pregnancies with isolated fetal CHD had a significantly higher incidence of adverse obstetric outcomes, including preeclampsia, preterm birth, or placental abruption [4, 10]. This relationship between the placenta and fetal heart development has been recapitulated in genetic murine models, providing further evidence that placental pathologies could be a primary cause of CHDs [12].

Despite these studies, there is a limited understanding of the placenta's role in cardiac development. Previous studies investigating placental pathology in pregnancies affected by fetal growth restriction often excluded fetal CHD. This study addresses this gap by evaluating placental pathology in CHD‐affected pregnancies. The study objectives were to compare the incidence of placental vascular pathologies in CHD compared to unaffected controls and to compare the incidence across CHD groups categorized by direction and oxygenation level of placental venous return streaming toward the fetal brain (e.g., left ventricular outflow tract obstruction, right ventricular outflow tract obstruction, and mixed). This physiology‐based approach enhances our understanding of the role of the placenta in the etiology of CHD, as we hypothesize that distinct placental pathologies are more prevalent among specific CHD subtypes due to differences in the preferential distribution of oxygenated venous return within the fetal circulation [13, 14].

## 2 Methods

### 2.1 Study Subjects

This multicenter, retrospective cohort study included pregnancies diagnosed with fetal CHD from 2016 to 2023 in Houston, Texas, and Boston, Massachusetts. The inclusion criteria were singleton pregnancies diagnosed with CHD, excluding patent foramen ovale and patent ductus arteriosus. Other exclusion criteria were genetic syndromic diagnosis, prematurity (less than 37 weeks' gestational age), and missing placental pathology reports. Placental data from unaffected pregnancies were obtained from Massachusetts General Hospital (*n* = 122) from uncomplicated singleton pregnancies. Exclusion criteria were similar to those of the study group, with the additional exclusion of pregnancies affected by fetal anomalies and growth restriction, as previously described [15]. CHD phenotype, genetic test results, delivery data, placental pathology results, and maternal and offspring demographics were collected. CHDs were categorized in accordance with the classification system described by the National Birth Defect Prevention Study [16]. Maternal pregnancy complications were also collected, including diabetes (pregestational or gestational), gestational hypertension, chronic hypertension, pre‐eclampsia with and without severe features, superimposed pre‐eclampsia with severe features, eclampsia, and hypertensive disorder of pregnancy that was not otherwise specified.

### 2.2 Congenital Heart Disease Classification

**FIGURE 1:**
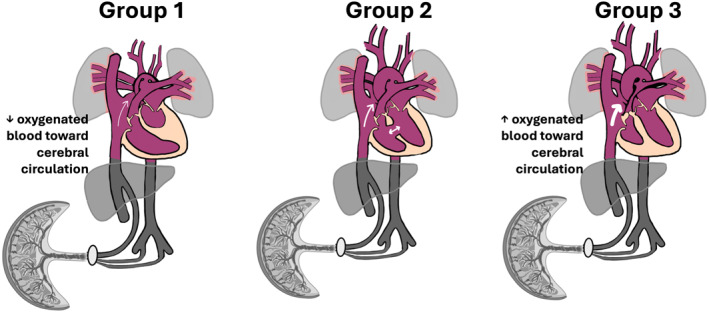
Congenital heart disease groups were based on preferential distribution of oxygenated blood toward fetal cerebral circulation. Group 1 (reduced preferential streaming of oxygenated blood toward fetal cerebral circulation) includes left ventricular outflow tract obstruction lesions, including hypoplastic left heart syndrome, coarctation of the aorta, aortic stenosis, transposition of great arteries, and Shone syndrome. Group 2 (intermediate preferential streaming of oxygenated blood toward fetal cerebral circulation due to intracardiac mixing) includes septal defects, tetralogy of Fallot, double outlet right ventricle, and truncus arteriosus. Group 3 (increased preferential streaming of oxygenated blood toward fetal cerebral circulation) includes right ventricular outflow tract obstruction lesions, including tricuspid atresia, pulmonary atresia or stenosis, and Ebstein anomaly. Created in Biorender.com.

CHD subtype were categorized into three groups based on the previously described expected patterns of preferential distribution of oxygenated venous return of placental blood within fetal circulation [17]. The classification proposed by Stanek is based on fetal brain blood supply found to correlate with placental pathology in left heart obstructive malformations [17]. This stratification is further supported by studies analyzing fetal neurodevelopment in CHD, which have demonstrated that the degree of preferential streaming of oxygenated blood toward fetal cerebral circulation varies by CHD subtype and influences neurodevelopmental outcomes [13, 14]. Therefore, these classifications are influenced by the underlying structural heart defect, as illustrated in Figure 1. Group 1 (reduced fetal cerebral oxygenation) includes left ventricular outflow tract obstruction lesions, such as hypoplastic left heart syndrome, coarctation of the aorta, aortic stenosis, transposition of great arteries, and Shone syndrome. In these lesions, oxygenated placental blood is preferentially directed toward the right ventricle and pulmonary artery, often with altered or reversed flow in the aortic arch, thereby reducing the proportion of oxygenated blood reaching the cerebral circulation. Group 2 (intermediate fetal cerebral oxygenation) includes septal defects, tetralogy of Fallot, double outlet right ventricle, and truncus arteriosus. These types of lesions result in intracardiac mixing of blood. Group 3 (increased fetal cerebral oxygenation) includes right ventricular outflow tract obstruction lesions, such as tricuspid atresia, pulmonary atresia or stenosis, and Ebstein anomaly. In these lesions, altered right‐sided flow dynamics may increase preferential streaming of oxygenated placental blood across the foramen ovale toward the left‐sided circulation and cerebral perfusion. The classification of reduced, intermediate, and increased cerebral oxygenation reflects differences in the preferential streaming of oxygenated placental blood in fetal intracardiac circulation.

### 2.3 Placental Pathology

In accordance with general obstetric practice, the physician determined placentas that required pathologic evaluation based on clinical indications, such as pregnancy loss, maternal infection, fetal growth restriction, preterm birth, non‐reassuring fetal heart tones, pre‐eclampsia with severe features, or neonates with evidence of multiorgan failure [15]. As such, placental pathology was not universally examined in all patients. Gross examination included the evaluation of the umbilical cord, placental disk, and membranes. Trimmed placental weight was measured after removal of the umbilical cord, fetal membranes, and nonadherent blood clots. Gross pathologies included placentas that were small for gestational age (< 10th percentile), large for gestational age (LGA; > 90th percentile), single umbilical artery, hypercoiled umbilical cord, abnormal cord insertion, and abnormal placental disk shape. Placental disks were sectioned at 1–2‐cm intervals to identify lesions within the parenchyma. Representative sections of the umbilical cord, placental parenchyma, and fetal membranes were examined. Abnormalities revealed by sectioning were included in the histological study. Placental pathology was evaluated in accordance with the Amsterdam Placental Workshop Group Consensus Statement, which identifies four principal patterns of placental injury: maternal vascular malperfusion (MVM), fetal vascular malperfusion (FVM), villitis of unknown etiology, and intrauterine infection [18]. Acute chorioamnionitis was also documented and subtyped into maternal inflammatory response or fetal inflammatory response. MVM included diagnoses of accelerated villous maturation, distal villous hypoplasia, decidual arteriopathy, infarct of > 5% of parenchyma, increased syncytial knots, and/or hemorrhage or hematoma. FVM included diagnoses of thrombosis anywhere within the fetal vascular tree, including intramural fibrin deposition, avascular villi, and venous ectasia. Villitis of unknown etiology included those with low‐grade and high‐grade villitis with or without avascular villi. Other lesions noted, if present, included delayed villous maturation and villous edema.

Placental pathology was classified according to the Amsterdam criteria in both CHD and unaffected control cases, as this classification had been adopted by pathologists at both institutions. Rates of placental pathology were compared between pregnancies complicated by CHD and unaffected patients, and across the three CHD groups. Continuous variables were analyzed using the Kruskal–Wallis rank‐sum test, and categorical variables were compared using Pearson's chi‐squared test or Fisher's exact test, as appropriate. A logistic regression model was used to assess perinatal factors associated with placental pathology among CHD cases with complete data (*n* = 417). A *p*‐value < 0.05 was considered the threshold for statistical significance. Measures of central tendency are presented as the median with the interquartile range (IQR).

## 3 Results

**FIGURE 2:**
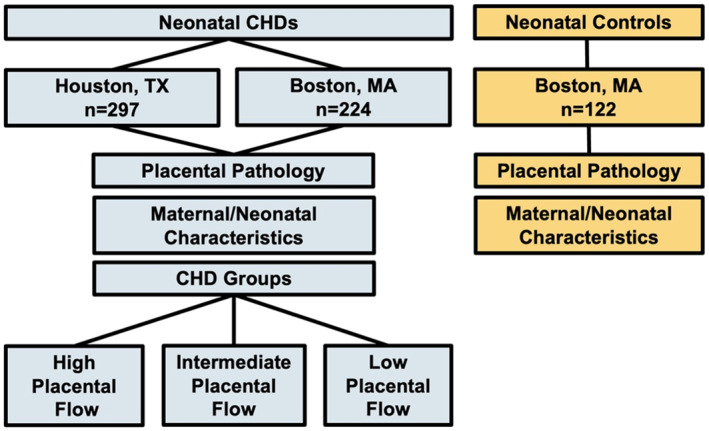
Summary of the two study cohorts. CHD = Congenital heart disease.

**FIGURE 3:**
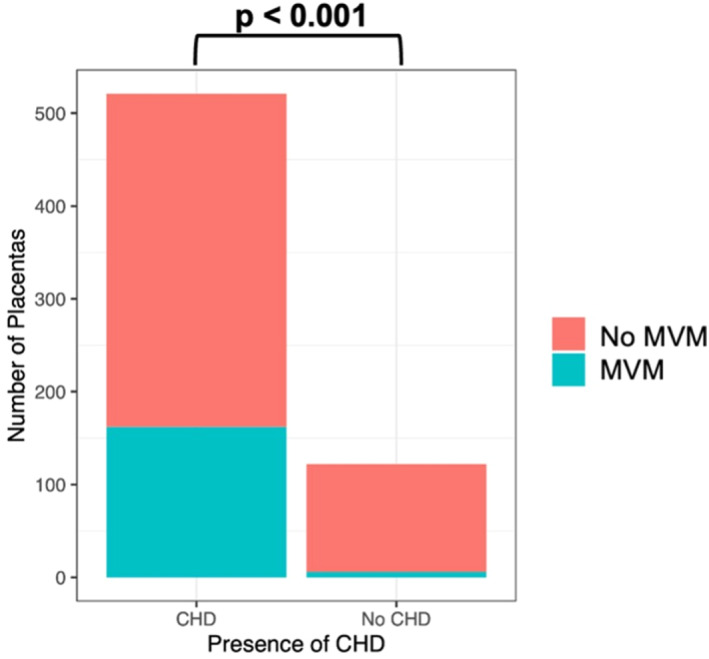
Comparison of the overall CHD population with the control population revealed higher rates of pathological patterns of MVM (0.31 vs. 0.05, *p* < 0.001). CHD = Congenital heart disease; MVM = Maternal vascular malperfusion.

In total, 521 pregnancies affected by CHD and 122 unaffected pregnancies were included in the study (Figure 2). The overall clinical characteristics of the cohorts are shown in Table 1. Overall, there was a significantly higher rate of MVM in the placentas from CHD pregnancies compared with the control population (0.31 vs. 0.05, *p* < 0.001) (Figure 3). Between CHD and control pregnancies, there were statistically significant differences in gestational age, placental hypoplasia, and MVM.

**TABLE 1:** Patient characteristics by fetal diagnosis.

Characteristics^a^	CHD (*n* = 521)	Control (*n* = 122)	*p*‐value
Gestational age, completed weeks, median [IQR]	38.00 [37.00, 39.00]	39.00 [38.00, 40.00]	**< 0.001**
Birth weight, grams, median [IQR]	3000 [2,630, 3385]	—	—
Male sex, *n* (%)	307 (59)	—	> 0.9
CHD category, *n* ^c^ (%)^d^			**< 0.001**
Group 1	240 (58)	0 (0)	—
Group 2	128 (31)	0 (0)	—
Group 3	49 (12)	0 (0)	—
Placental weight, grams, median [IQR]^b^	466 [390, 547]	460 [410, 550]	> 0.9
Placental hypoplasia, *n* (%)^b^	63 (28)	49 (40)	**0.022**
Placental MVM, *n* ^b^ (%)^d^	162 (31)	6 (4.9)	**< 0.001**
Placental FVM, *n* ^b^ (%)^d^	48 (9.3)	11 (9.0)	> 0.9
Maternal age, years, median [IQR]	31 [26, 36]	—	—

*Note:* Bold values indicate statistical significance (*p* < 0.05).

^a^ Data presented as median [IQR] or *n* (%).

^b^ Placental characteristics were assessed only in pregnancy with completed placental pathology reports; percentages reflect the number of affected cases divided by the number with available data.

^c^ CHD categories were calculated among 417 cases, which represents the number of cases with complete CHD classification and placental pathology data.

^d^ CHD = congenital heart disease; MVM = maternal vascular malperfusion; FVM = fetal vascular malperfusion.

Placental pathology and clinical characteristics were compared among the three CHD groups (Table 2). Maternal and neonatal characteristics, including gestational age, birth weight, sex, and maternal age, significantly differed among the groups. Placental MVM was present in approximately 30% of all three CHD groups. However, FVM pathology was more common in left ventricular outflow tract lesions in the group with reduced fetal cerebral oxygenation (*p* = 0.037).

**TABLE 2:** Patient characteristics by CHD group and associated placental pathology.

Characteristics^a^	Group 1 (*n* = 240)	Group 2 (*n* = 128)	Group 3 (*n* = 49)	*p*‐value
Gestational age, weeks, median [IQR]	38.00 [37.00, 39.00]	38.00 [37.00, 39.00]	37.00 [37.00, 39.00]	**0.007**
Birth weight, grams, median [IQR]	3100 [2,718, 3500]	2950 [2,455, 3283]	2975 [2,570, 3450]	**0.002**
Male sex, *n* (%)	84 (35)	68 (53)	15 (31)	**< 0.001**
Placental weight, grams, median [IQR]^b^	474 [394, 538]	455 [378, 543]	490 [415, 578]	0.2
Placental hypoplasia, *n* (%)^b^	38 (28)	12 (38)	3 (14)	0.2
Placental MVM, *n* ^b^ (%)^c^	69 (29)	44 (34)	15 (31)	0.5
Placental FVM, *n* ^b^ (%)^c^	24 (10)	11 (8.6)	0 (0)	**0.037**
Maternal age, years, median [IQR]	32 [27, 36]	30 [24, 34]	33 [28, 37]	**0.014**

*Note:* Bold values indicate statistical significance (*p* < 0.05).

^a^ Data presented as median [IQR] or *n* (%).

^b^ Placental characteristics were assessed only in pregnancy with completed placental pathology reports; percentages reflect the number of affected cases divided by the number with available data.

^c^ MVM = maternal vascular malperfusion; FVM = fetal vascular malperfusion.

We also compared the four Amsterdam placental criteria against maternal and neonatal clinical characteristics in a logistic regression model among CHD cases with complete data (*n* = 417), including FVM (*n* = 35), MVM (*n* = 128), and villitis of unknown etiology (*n* = 59). MVM was associated with low birth weight (OR = 0.28, *p* < 0.001) (Table 3). The birth weight of infants with placental MVM was lower than that of infants without placental MVM (median 2.7 vs. 3.1 kg, *p* = 1.08 × 10^−9^). Increased maternal age was a significant predictor of FVM (OR = 1.07, *p* = 0.037) (Table 4). No significant associations were identified in villitis of unknown etiology or intrauterine infection.

**TABLE 3:** Associations of MVM with neonatal and maternal characteristics.

Characteristics^a^	OR	95% CI	*p*‐value
Birth weight	0.28	[0.16, 0.48]	**< 0.001**
Gestational age	1.07	[0.90, 1.27]	0.4
Male sex	0.76	[0.47, 1.21]	0.2
CHD group 1	—	—	—
CHD group 2	0.97	[0.58, 1.63]	> 0.9
CHD group 3	0.89	[0.41, 1.87]	0.8
Maternal age	0.98	[0.94, 1.01]	0.2

*Note:* Bold values indicate statistical significance (*p* < 0.05).

^a^ OR = odds ratio; CI = confidence interval; MVM = maternal vascular malperfusion; CHD = congenital heart disease.

**TABLE 4:** Associations of FVM with neonatal and maternal characteristics.

Characteristics^a^	OR	95% CI	*p*‐value
Birth weight	0.47	[0.17, 1.16]	0.12
Gestational age	1.09	[0.81, 1.50]	0.6
Male sex	1.33	[0.56, 3.33]	0.5
CHD group 1	—	—	—
CHD group 2	0.43	[0.14, 1.15]	0.12
CHD group 3^b^	—	—	> 0.9
Maternal age	1.07	[1.00, 1.14]	**0.037**
Hypertension	1.41	[0.53, 3.47]	0.5

*Note:* Bold values indicate statistical significance (*p* < 0.05).

^a^ OR = odds ratio; CI = confidence interval; FVM = fetal vascular malperfusion; CHD = congenital heart disease.

^b^ No events occurred in CHD Group 3; OR and CI could not be calculated.

## 4 Discussion

This study provides supporting evidence that placental pathology is more prevalent in CHD‐affected pregnancies. These findings expand current models of the placenta‐heart axis by demonstrating that specific patterns of placental dysfunction are associated with certain CHD subtypes, stratified by the degree of preferential streaming of oxygenated venous return toward the fetal brain [7]. This framework extends the placenta‐heart axis to a placenta‐heart‐brain axis, recognizing that disruptions in oxygenated venous return may simultaneously drive placental dysfunction and adverse neurodevelopmental outcomes in CHD‐affected pregnancies [19]. Applying this framework to placental pathology allows us to explore whether these hemodynamic patterns are reflected in distinct placental histopathology. Altered fetal hemodynamic patterns in the setting of fetal CHD may not only result from cardiac structural abnormalities but may also contribute bidirectionally to abnormal placental development, further perpetuating the placenta‐heart‐brain developmental axis.

Our findings demonstrate that MVM is associated with CHD, aligning with existing scientific knowledge [19, 20, 21]. The rate of FVM was inversely related to the group of CHDs associated with reduced fetal cerebral oxygenation, which includes transposition of the great arteries and other defects with diminished streaming of oxygenated blood toward the fetal brain. However, it is important to note that the grouping system employed in this study did not directly measure cardiac output or cerebral perfusion. Although specific lesions, such as hypoplastic left heart syndrome, are associated with reduced cardiac output and altered cerebral perfusion, these parameters were not directly assessed due to limitations inherent to routine fetal echocardiography [22]. Accordingly, our findings should be interpreted as associative rather than mechanistic. Increased FVM (fetal side) may reflect downstream consequences of altered fetal circulatory streaming, whereas MVM (maternal side) may represent upstream maternal contributions to placental pathology. Together, these findings are consistent with a bidirectional relationship between placental and cardiac development and emphasize the value of placental pathology in refining disease associations in CHD.

Maternal and neonatal characteristics, including gestational age, birth weight, sex, and maternal age, significantly differed among the various patterns of placental injury. MVM was positively correlated with low birth weight, suggesting that placental perfusion defects are associated with low‐normal estimated fetal weight (Table 3). MVM refers to disruptions in maternal blood flow to the placenta, commonly caused by pre‐eclampsia or thrombophilias, and is often characterized by placental infarctions. This leads to chronic fetal hypoxia and nutrient deprivation, which is a primary contributor to fetal growth restriction. Several studies have shown that the presence and increasing severity of MVM lesions are associated with a higher risk of fetal growth restriction and lower mean birth weight, even in the absence of maternal disease [23, 24].

FVM was significantly associated with increased maternal age, suggesting that in the aged placenta, there is a higher occurrence of placental lesions that impair blood flow from the fetus throughout the placenta. FVM is often caused by abnormalities in fetal blood flow within the placenta, resulting from obstruction or stasis in fetal vessels, such as from umbilical cord compromise or thrombosis. Histopathologic features include thrombi in fetal vessels, avascular villi, or villous stromal fibrosis [25, 26]. Advanced maternal age, defined as greater than 35 years, is associated with an increased risk of placental dysfunction, including FVM. Advanced maternal age is also independently associated with a higher risk of CHD in the fetus [27].

Radford et al. previously revealed that genetic defects in the syncytiotrophoblast layer, the outermost placental layer directly exposed to maternal blood, can cause CHD in offspring [12]. Our findings, which reveal significant differences in MVM when comparing unaffected controls to CHD and FVM across CHD groups, highlight the importance of both maternal and fetal circulatory integrity for placental health. Altered fetal hemodynamic patterns and preferential distribution of oxygenated blood in CHD‐affected pregnancies may impair placental function, especially the syncytiotrophoblast layer, contributing to a cycle of compromised exchanges that influences fetal cardiovascular development. Leon et al. reported that vascular malperfusion lesions and chronic forms of inflammation occur at higher rates in placentas complicated by CHD, suggesting fetal congenital malformations can negatively impact placental function. It was also described that placentas from pregnancies with fetal CHD had increased rates of villitis of unknown etiology [28]. In our study, villitis of unknown etiology was not significantly associated with CHD. However, the causal relationship, whether placental dysfunction drives CHD development or vice versa, remains unclear. Similarly, O'Hare et al. showed that placentas of fetal CHD exhibited high rates of delayed villous maturation and MVM but not FVM [29]. Although the study was limited by a smaller sample size, it also utilized the Amsterdam criteria for placental pathology classification. Recently, Carreon et al. reported that the prevalence of vascular malperfusion or dysfunction did not differ by fetal CHD type in a smaller cohort [30]. Our study distinguishes itself from prior studies by grouping CHD types according to previously described patterns of preferential streaming of oxygenated blood toward fetal cerebral circulation, which is critical given the heterogeneity of CHD. Additionally, we were able to compare the placental pathology of CHD groups to unaffected placentas, which was not performed in the original Stanek study, allowing for a more complete characterization of placental pathology across CHD groups [17].

Several potential mechanisms could explain our results. MVM rates were consistent across all three CHD groups, suggesting that maternal blood supply to the placenta plays a crucial role in CHD pathogenesis across various subtypes. This finding highlights the importance of maternal vascular insufficiency, as restricted maternal blood flow could lead to fetal hypoxia and nutrient deprivation, which are key environmental stressors that may disrupt fetal cardiovascular development. Additionally, FVM was significantly more frequent in left ventricular outflow tract defects (Group 1), characterized by reduced preferential streaming of oxygenated blood toward fetal cerebral circulation. Altered intracardiac streaming patterns may reduce the proportion of oxygenated blood preferentially directed toward the cerebral circulation, which may contribute to impaired neurodevelopment as suggested in prior studies [31]. FVM lesions may reflect altered fetal hemodynamic patterns associated with these structural abnormalities rather than solely secondary placental dysfunction [32]. These placental lesions are associated with an increased risk of fetal growth restriction, stillbirth, and neurodevelopmental injury [33]. Therefore, understanding FVM patterns in CHD may inform future strategies for prenatal surveillance and risk stratification.

Given the central role of vascular development in both the placenta and fetal heart, angiogenic signaling represents a plausible biological pathway linking these processes. Dysregulation of key angiogenic mediators, including vascular endothelial growth factor and placental growth factor, has been implicated in abnormal placental vascularization and impaired cardiac morphogenesis. Emerging evidence demonstrates that imbalances between angiogenic and anti‐angiogenic factors are associated with both placental pathology and CHD, supporting a shared mechanistic framework underlying these conditions [34]. Further investigation into angiogenic signaling pathways may therefore clarify common developmental mechanisms connecting placental dysfunction and CHD.

This study provides valuable insight into the association between placental vascular pathology and CHD. A key strength of this study is the inclusion of two large academic institutions that provide specialized deliveries for pregnancies affected by fetal CHD along with neonatal intensive care with a control comparison group, enhancing the robustness and generalizability of our findings. Our study adhered to the Amsterdam criteria, ensuring standardized placental sampling and consistent diagnosis. It is important to note that a subset of the fetal CHD subjects in our study was previously analyzed by the Boston group [30]. However, our cohort includes twice as many cases, drawn from two institutions, and classifies fetal CHD based on fetal‐placental physiology rather than the prior schema. The concordance in MVM incidence across both studies strengthens and validates our findings as well as those reported previously. Another strength of our study lies in our nuanced approach to preeclampsia, a condition associated with CHD but characterized by variable clinical definitions and diagnostic criteria. Rather than relying on clinical diagnosis, which carries inherent uncertainty, we evaluated placental pathology directly, reducing potential bias. Specifically, we focused on MVM, a key histopathologic feature of preeclampsia. MVM is not only central to the diagnosis of pre‐eclampsia in the Amsterdam criteria, but it has also been shown to correlate strongly with clinical outcomes. In a prospective study of low‐risk nulliparous women, MVM lesions were associated with a 4.5‐fold increased risk of developing pre‐eclampsia or delivering a small‐for‐gestational‐age neonate [35]. Therefore, our study more precisely identified vascular insufficiency relevant to fetal cardiac development and growth restriction rather than relying on clinical diagnosis.

Limitations of the study include a relatively small sample size within the CHD groups. While there was a large overall CHD study population, the groups were unbalanced, particularly in right ventricular outflow tract obstruction lesions (Group 3). This imbalance may have influenced our findings and should be considered when interpreting results. We also acknowledge the inherent complexity in classifying CHDs, particularly when anatomic variations overlap across diagnostic boundaries. To enhance consistency and comparability with previous population‐based studies, we used the Level 2 categorization from the National Birth Defect Prevention Study [16]. Additionally, FGR was excluded from the control group, resulting in a highly normalized comparison group. An optimal study design would include a control group of structurally normal fetuses with a naturally occurring prevalence of FGR to isolate the sole contribution of CHD to placental abnormalities. The retrospective nature of this study limits causal inference. Placental pathology in both the control and CHD groups was clinically indicated rather than universally collected, which may introduce selection bias and limit the generalizability of the findings.

## 5 Conclusion

Overall, our study advances the understanding of the relationship between placental dysfunction and CHD. These findings support the need for increased monitoring of placental health in pregnancies affected by CHD. Future research should investigate angiogenic factors as potential mediators of this relationship, helping to elucidate the mechanisms by which placental circulation influences fetal cardiac development. A deeper understanding of the molecular pathways underlying the placenta‐heart axis could inform targeted interventions and preventative strategies aimed at improving placental health, with the ultimate goal of reducing CHD risk and improving maternal and fetal outcomes.

## Funding

The authors have nothing to report.

## Ethics Statement

This study was approved by the UTHealth Houston Institutional Review Board on April 22, 2024 (Approval Number: HSC‐MS‐24‐0210) and was determined to qualify for exempt status under 45 CFR 46.104(d), Category #4.

## Consent

The authors have nothing to report.

## Conflicts of Interest

The authors declare no conflicts of interest.

## Data Availability

The data that support the findings of this study are available from the corresponding author upon reasonable request.
